# Gout, flares, and allopurinol use: a population-based study

**DOI:** 10.1186/s13075-019-1918-7

**Published:** 2019-05-31

**Authors:** Charlotte Proudman, Susan E. Lester, David A. Gonzalez-Chica, Tiffany K. Gill, Nicola Dalbeth, Catherine L. Hill

**Affiliations:** 1 Adelaide Medical School, Faculty of Health and Medical Sciences, The University of Adelaide, Adelaide, South Australia; 2 Rheumatology Unit, The Queen Elizabeth Hospital, Woodville Road, Woodville South, 5011 South Australia; 3 Discipline of Medicine, Faculty of Health and Medical Sciences, The University of Adelaide, Adelaide, South Australia; 4 Discipline of General Practice, Faculty of Health and Medical Sciences, The University of Adelaide, Adelaide, South Australia; 5 Department of Medicine, University of Auckland, Auckland, New Zealand

**Keywords:** Gout, Gout flares, Allopurinol, Self-reported, Prevalence, Population study

## Abstract

**Background:**

There is a paucity of community-based data regarding the prevalence and impact of gout flares as these may often be self-managed. The aim of this study was to determine the prevalence of self-reported gout and gout flares, the use of urate-lowering therapy (ULT), and the association of gout flares with health-related quality of life (HRQoL) in a large community sample. Covariate associations with flare frequency and allopurinol use were also examined.

**Methods:**

The South Australian Health Omnibus Survey is an annual, face-to-face population-based survey. Data collected in the 2017 survey included self-reported medically diagnosed gout, allopurinol use (first-line ULT in Australia), and gout attacks (flares) in the last 12 months, in addition to sociodemographic variables and health-related quality of life (HRQoL, SF-12). Data were weighted to the Australian Bureau of Statistics 2016 census data to reflect the South Australian population. Participants 25 years and over (*n* = 2778) were included in the analysis.

**Results:**

The prevalence of gout was 6.5% (95%CI 5.5, 7.5). Amongst participants with gout, 37.1% (95%CI 29.6, 45.3) reported currently using allopurinol, while 23.2% (95%CI 16.9, 21.0) reported prior use (38% discontinuation rate). Frequent flares (≥ 2 in the last year) were reported by 25% of participants with gout and were more likely with younger age, higher body mass index, and current allopurinol use (*p* < 0.05). The frequency of gout flares was associated with a lower physical HRQoL (*p* = 0.012). Current allopurinol use was reported by 51% of participants with frequent gout flares.

**Conclusion:**

Flares were frequently reported by people with gout in the community**.** Gout flares were associated with reduced physical HRQoL. Almost one half of people with frequent gout flares were not receiving allopurinol, and current allopurinol use was associated with frequent gout flares, suggesting undertreated disease and suboptimal use of ULT. Determining covariate associations with flares and ineffective allopurinol use may identify means of improving treatment and reducing flares.

**Electronic supplementary material:**

The online version of this article (10.1186/s13075-019-1918-7) contains supplementary material, which is available to authorized users.

## Background

Gout is the most common inflammatory arthritis. The reported prevalence of gout is highly variable across the world, ranging from 0.1% to approximately 10%, with prevalence estimates greater than 1% in most developed countries [1]. The prevalence of gout in the UK was recently estimated as 2.5% [2], 3.9% in the USA, [3], and 5.2% in a recent Australian cohort study [4]. High baseline serum urate level and subcutaneous tophi have been linked to increased mortality, mostly attributable to cardiovascular disease [5, 6]. Furthermore, despite advances in understanding of the pathophysiology, risk factors, and therapy, gout remains a burden on the individual’s health-related quality of life (HRQoL) and on healthcare resources [7–9].

Current guidelines from the American College of Rheumatology (ACR) and the European League Against Rheumatism (EULAR) advise that long-term urate-lowering therapy (ULT), with the aim of maintaining serum urate levels (generally below 6 mg/dL) [10, 11], is key to effective control of gout and should be initiated in the presence of certain clinical features: for example, tophi, frequent gouty attacks (flares; two or more per year), and urate arthropathy [12].

Nonetheless, studies suggest a poor adherence to guidelines [13–16]. Reasons for this include inappropriate ULT dosing by prescribers or inadequate monitoring of serum urate levels [16, 17] and low rates of continuation of therapy when prescribed [18]. Gout flares are a clinical indicator of disease severity and the need for commencing or optimizing ULT and may continue to occur in up to one third of patients [19–21].

There is a paucity of community-based data regarding gout flares. One internet-based case-crossover US-based study found that 53% of enrolled participants did not consult a health care physician during an acute gout flare [22], suggesting most gout flares are self-managed in the community and that the “treatment gap” may be under-estimated.

The aim of this study was to identify the prevalence of self-reported gout flare and the frequency of allopurinol use in a representative community-based survey. In addition, sociodemographic and clinical covariates associated with flare frequency and associations with comorbidities and HRQoL were sought.

## Methods

### Study population

Data were obtained from the 2017 South Australian Health Omnibus Survey (HOS). The HOS is an annual, population survey, conducted by face-to-face interviews of approximately 3000 people aged 15 years and over, that obtains cross-sectional representative information on health, well-being, and related issues amongst the South Australian population living in metropolitan and rural areas. HOS has been designed to meet the highest standards of population survey methodology and is a clustered, multi-stage, systematic, self-weighting area sample [23, 24].

The 2017 HOS survey consisted of data from 2977 interviews from 5300 selected households (participation rate 65.3%) and was conducted between September and December 2017.

### Outcomes

Within the survey interview, three gout-related questions were asked, “Have you even been told by a doctor that you have gout?” with the response options of “Yes,” “No,” or “Do not know/refused.” To determine allopurinol use, the respondents were asked “Do you currently take/have you taken allopurinol for gout?” with the response options of “No, never taken” (never), “No, previously taken” (prior), or “Yes, still taking” (current), with a list of the current brand names of allopurinol available to the interviewees. Febuxostat was not included because it only became available on the Australian Pharmaceutical Benefits Scheme in 2015 as a second-line option if allopurinol was contraindicated or not tolerated [25]**.** A recent US study reported little change to gout therapy since the introduction of febuxostat to the market (prescribed to only 3% of gout study population) [26].

To determine frequency of flares, respondents were asked, “If you have gout, how many gout attacks have you had over the last 12 months?” with the response options comprising of “None,” “One,” “Two,” “Three,” “Four,” or “Five or more.”

### Covariates

Sociodemographic data collected included age, gender, and socioeconomic status (SES). SES was determined using the Index of Relative Socioeconomic Advantage and Disadvantage (IRSAD) [27], which is normalized to a mean of 1000 and standard deviation of 100, and where a low index score suggests relative disadvantage, and a higher index score represents relative advantage.

Body mass index (BMI) was based on self-reported height and weight, calculated according to standard formula, and was classified according to World Health Organization (WHO) criteria [28]. Information on comorbidities was obtained from the questions “Has a doctor ever told you that you have: (a) a heart attack/angina or did you undergo a heart procedure to unblock blocked vessels in your heart (called angioplasty or stenting), (b) Stroke, (c) High blood pressure, (d) Diabetes/high blood sugar, (e) High cholesterol levels”.

HRQoL was measured by the SF-12 v1 (US version) with Physical Component Scores (PCS) and Mental Component Scores (MCS) computed as norm-based *t*-scores with a mean of 50 and a standard deviation of 10 [29].

### Statistical analyses

Analyses were performed using Stata v15.1 (StataCorp LLC, Texas, USA), and all tabulations, descriptive statistics, and regression models utilized appropriate survey weights. Only participants aged 25 and over were included in the analysis because gout is a disease of older adults. Prior to analysis, flares were grouped into three categories: none, 1, and ≥ 2 because, according to the ACR, ULT is indicated in patients with two or more gout flares/year [12].

Data were weighted by the inverse of the individual’s probability of selection, as well as the response rate in metropolitan and country regions and then re-weighted to benchmarks derived from the Australian Bureau of Statistics 2016 Census data (age and sex). These person weights adjust the data to better align each case (individual) with the age, gender, and geographic location distribution in the total South Australian population. Survey-weighted logistic (gout prevalence), multinomial logistic (allopurinol use and frequency of gout flares per year), and linear (HRQoL) regression models were used to analyze relationships with relevant predictor variables. All models included the sociodemographic variables of age, gender, SES (IRSAD score), and BMI, with both linear and quadratic regression terms to allow for non-linearity in any relationship with the response variable. To enable interpretation of regression models, Stata post estimation commands were used to express results for each outcome as adjusted population-weighted marginal proportions/probabilities (for logistic or multinomial models), or predicted means (for linear regression), for different levels of each predictor variable, and to determine the effect size (the derivative or change in the marginal outcome with a change in the predictor variable) averaged over other covariates. For multinomial outcomes (flares and allopurinol use), Helmert contrasts of the outcomes were used to define meaningful comparisons, and joint *p* values were reported.

## Results

### Gout prevalence and relationship to sociodemographic variables

Of the 2977 interviews conducted, 2778 participants, aged 25 years and over, were included in the analysis, with 71.4% (95% CI 69.3, 73.4) born in Australia and 10.0% (95% CI 8.9, 11.2) born in the UK. The estimated gout prevalence was 6.5% (95% CI 5.5, 7.5).

Comparison of sociodemographic variables (sex, age, and SES), BMI, and comorbidities between the general South Australian population and group with gout are presented in Table 1. Participants with gout were more likely to be male (*p* < 0.001), were older (*p* < 0.001), have a higher BMI (*p* < 0.001), and have a lower SES (*p* = 0.022, Additional file 1: Table S1). There was a high burden of comorbidities in participants with gout, including heart disease (24%), diabetes (33%), high blood pressure (54%), and high cholesterol (40%), which is consistent with their sociodemographic profile. The prevalence of gout by sex, by age group (decades), and by BMI (WHO classification) is reported in Additional file 1: Table S2, and the prevalence breakdown by sex and age group is reported in Additional file 1: Table S3.

**Table 1:** Sociodemographic variables in the entire South Australian study population and participants with gout^1,2^

Demographic	Entire SA population	Participants with gout
%Males	48.7 (46.5, 50.9)^2^	79.2 (72.4, 84.6)
Age: Mean	52.3 (51.5, 53.1)	63.3 (60.5, 66.0)
%25–34 years	18.2 (16.4, 20.2)	7.6 (3.7, 15.0)
%35–44 years	17.8 (16.1, 19.7)	6.6 (2.9, 14.3)
%45–54 years	19.4 (17.6, 21.4)	8.5 (5.0, 14.0)
%55–64 years	18.5 (16.9, 20.2)	22.8 (16.3, 31.0)
%65+ years	26.1 (24.3, 27.9)	54.5 (46.2, 62.6)
SES (IRSAD): Mean	970 (961, 978)	954 (938, 971)
BMI^3^: Mean	27.5 (27.2, 27.8)	30.3 (29.2, 31.4)
%Normal/Underweight	35.1 (32.9, 37.3)	15.5 (10.7, 21.9)
% Overweight	38.6 (36.4, 40.9)	40.1 (32.6, 48.2)
% Obese	26.3 (24.3, 28.4)	44.0 (36.5, 52.6)
SF-12:		
PCS: Mean	47.5 (47.0, 48.0)	42.5 (40.4, 44.6)
MCS: Mean	53.2 (52.8, 53.6)	53.9 (52.7, 55.0)
Comorbidities:		
% Heart attack/angina	8.1 (7.0, 9.2)	23.8 (17.6, 31.3)
%Heart failure	1.6 (1.2, 2.3)	3.6 (1.8, 7.1)
% Stroke	2.2 (1.8, 2.9)	3.7 (1.8, 7.3)
% High blood pressure	30.9 (29.0, 33.0)	53.9 (45.6, 61.9)
% Diabetes/high blood sugar	13.2 (11.8, 14.7)	32.8 (25.9, 40.5)
% High cholesterol	25.9 (24.0, 27.8)	40.1 (32.8, 47.9)

*SA* South Australia, *SES* socioeconomic status, *IRSAD* Index of Relative Socioeconomic Advantage and Disadvantage, *BMI* body mass index, *SF-12* Short Form (12 questions) Health Survey, *PCS* Physical Component Score, *MCS* Mental Component Score

^1^Aged 25 years and over

^2^Parentheses enclose 95% confidence intervals

### Allopurinol use by participants with gout

Current allopurinol was reported by 37.1% of the participants with gout, prior use by 23.2% (discontinuation rate of 38%), and 39.7% reported never using allopurinol (Table 2). Current allopurinol use amongst participants with gout was not associated with SF-12 PCS (Table 3) nor MCS.

**Table 2:** Two-way tabulation (%) of allopurinol use by flares in participants with gout

Allopurinol	Number of gout flares in the preceding year (%)^1^			Total
	None	1	≥ 2	
Never used	22.1 (16.3, 29.2)	10.0 (6.1, 15.9)	7.6 (4.1, 13.6)	39.7 (31.8, 48.2)
Prior use	14.5 (9.4, 21.8)	4.2 (1.8, 9.4)	4.5 (2.3, 8.6)	23.2 (16.9, 21.0)
Current use	21.6 (15.7, 28.9)	3.0 (1.3, 6.6)	12.5 (7.9, 19.2)	37.1 (29.6, 45.3)
Total	58.2 (50.3, 65.8)	17.2 (11.8, 24.3)	24.6 (18.3, 32.2)	100

^1^Percentages are absolute percentages of the entire gout subpopulation, and numbers in brackets are 95% confidence intervals

**Table 3:** SF-12 Physical Component Scores (PCS) by flares and allopurinol use in participants with gout

Predictor	SF-12 PCS	Effect size (difference)^1^	*p* value
Allopurinol			
Never used	43.6 (40.9, 46.3)^2^	Base	
Prior use	41.6 (37.7, 45.4)	− 2.0 (− 6.7, 2.7)	0.41
Current use	42.6 (39.9, 45.2)	− 1.0 (− 5.1, 3.1)	0.64
Joint			0.70
Number of gout flares in the preceding year			
None	44.7 (42.7, 46.8)	Base	
1	42.2 (38.3, 46.1)	− 2.6 (− 7.3, 2.2)	0.29
≥ 2	37.9 (34.0, 41.9)	− 6.8 (− 11.3, − 2.3)	0.003
Joint			0.012

^1^Analysis was performed by survey weighted multiple linear regression and results expressed as population-averaged estimates, adjusted for additional covariates age, gender, BMI, and socioeconomic status (IRSAD)

^2^Numbers in brackets are 95% confidence intervals

**Fig. 1:**
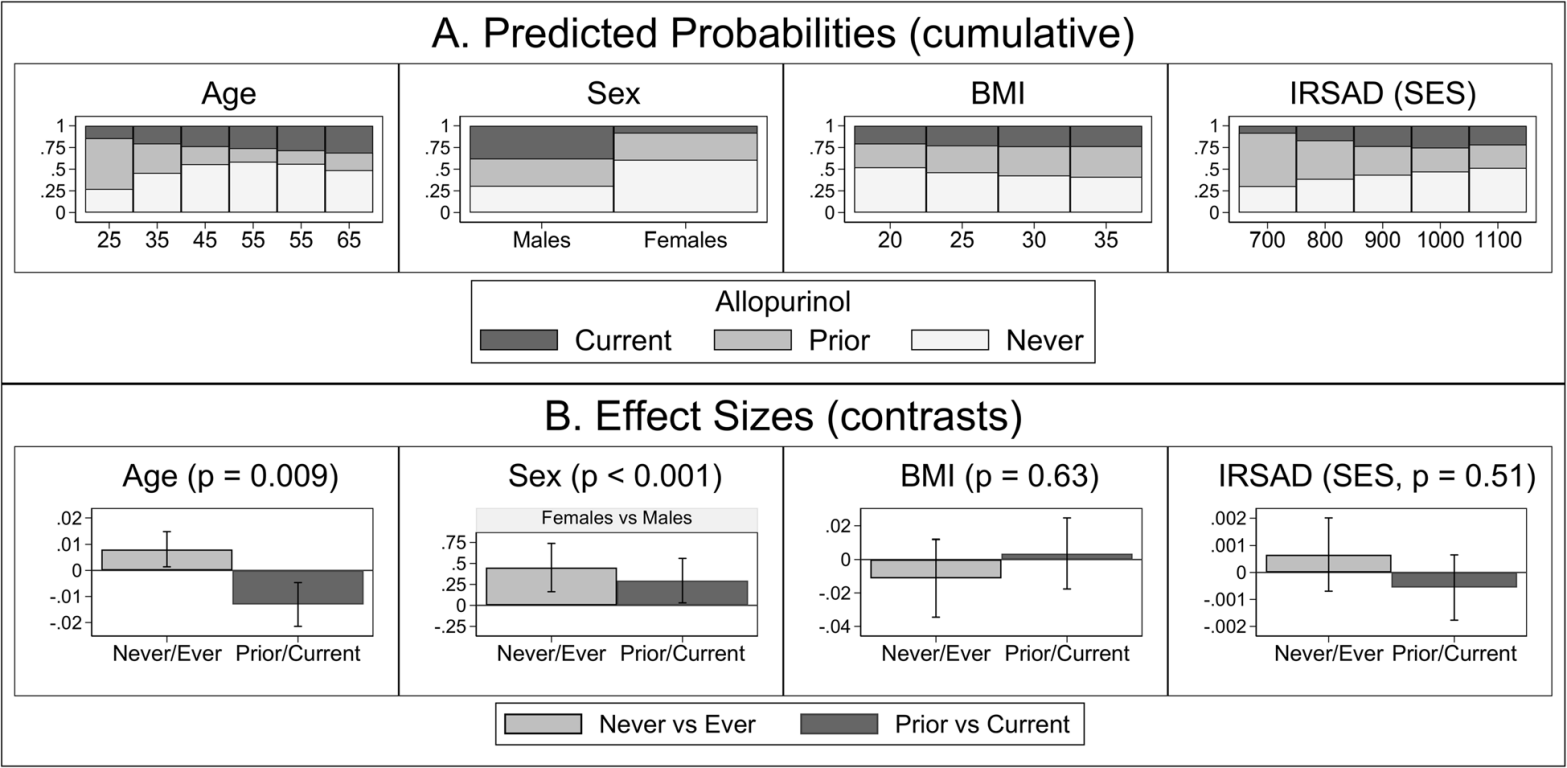
Covariates associated with allopurinol use in participants with gout. Legend: **a** Predicted, population-averaged marginal probabilities for each category of allopurinol use (classified as never, prior, current) use by covariates (stacked bar charts) and **b** risk difference effect sizes (outcome Helmert contrasts) for covariate associations with allopurinol use, with vertical bars representing 95% confidence intervals. Analysis was performed by multinomial logistic regression

Sociodemographic and clinical covariates of allopurinol use were analyzed by multinomial logistic regression (Additional file 1: Table S4), and the predicted outcome probabilities and effects sizes are depicted in Fig. 1. Age (*p* = 0.009) and sex (*p* < 0.001) were associated with allopurinol use, whereas BMI (*p* = 0.63) and SES (IRSAD, *p* = 0.51) were not. Older age was associated with a higher probability of allopurinol never-use (contrast 0.008, 95% CI 0.001, 0.015, *p* = 0.018), yet amongst allopurinol ever-users, a lower probability of discontinuation was observed (contrast − 0.013, 95% CI − 0.021, − 0.005, *p* = 0.002). Females were also associated with a higher probability of allopurinol never-use (contrast 0.45, 95% CI 0.16, 0.74, *p* = 0.002), as well as a higher probability of discontinuation (contrast 0.30, 95% CI 0.03, 0.56, *p* = 0.029) amongst allopurinol ever-users.

### Flares in participants with gout

A majority of the participants with gout reported no flares in the last 12 months (58%); however, nearly 25% reported having two or more flares during this time (Table 2). The adverse impact of flares on HRQoL is illustrated by an inverse ordinal relationship between increasing number of flares and decreasing SF-12 PCS (Table 3, *p*_linear trend_ 0.003). There was no such trend for SF-12 MCS (*p* = 0.74).

Only 51% (12.5/24.6) of participants with two or more flares/year were currently taking allopurinol (Table 2).

**Fig. 2:**
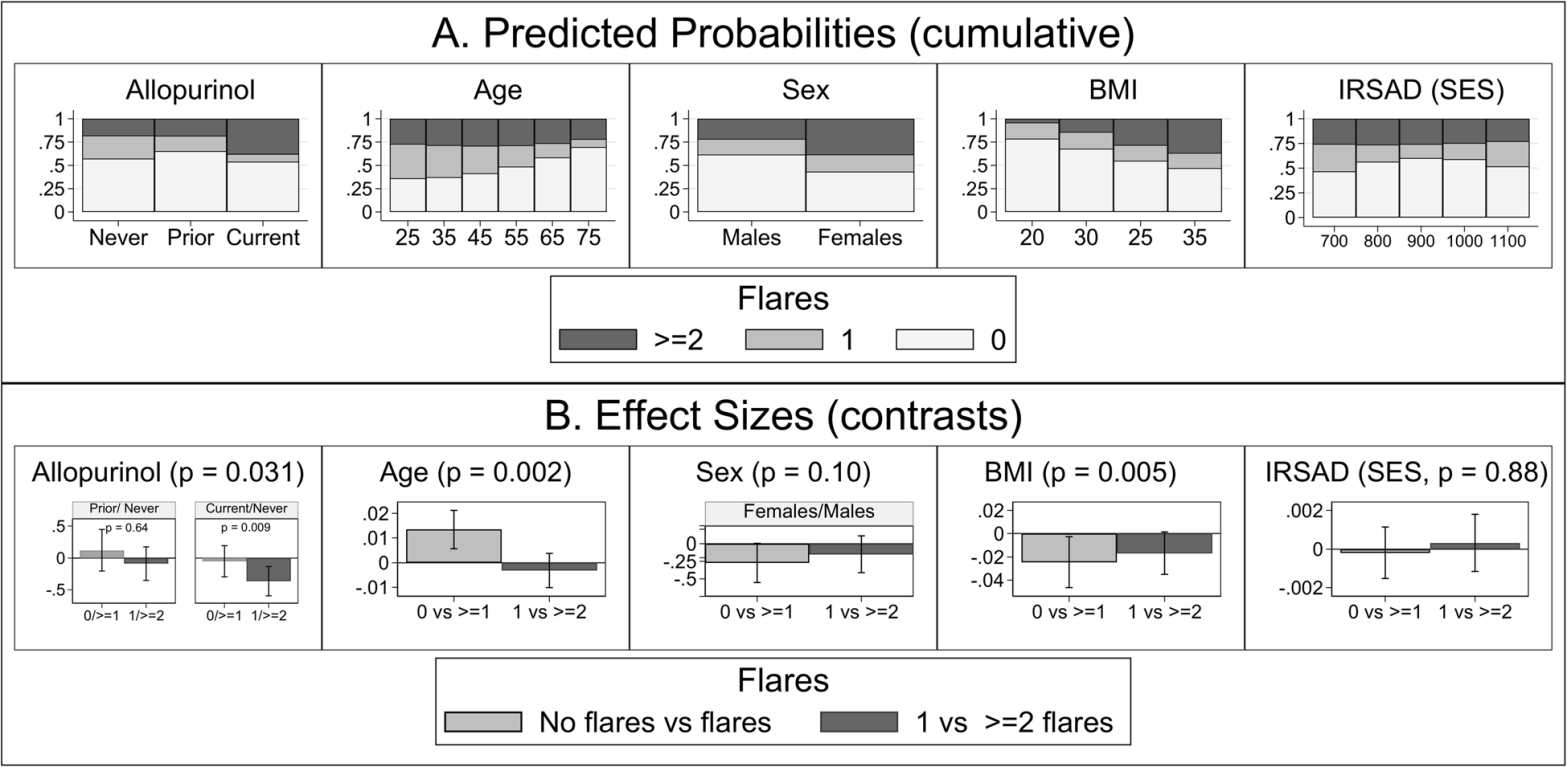
Covariate associations with flares in participants with gout. Legend: **a** Predicted, population-averaged marginal probabilities for each category of flares (classified as 0, 1, ≥ 2) by covariates (stacked bar charts) and **b** Risk difference effect sizes (outcome Helmert contrasts) for covariate associations with flares, with vertical bars representing 95% confidence intervals. Analysis was performed by multinomial logistic regression

The relationships between allopurinol use, sociodemographic and clinical variables, and flares were analyzed by multinomial logistic regression (Additional file 1: Table S5), and the predicted outcome probabilities and effect sizes are depicted in Fig. 2. Age (*p* = 0.002), BMI (*p* = 0.005), and allopurinol use (*p* = 0.031) were the most important covariates associated with flares. Older age was most strongly associated with a decreased probability of flares (contrast − 0.013, 95% − 0.021, − 0.006, *p* = 0.001). Although sex did not reach overall statistical significance (*p* = 0.10), possibly due to the relatively low proportion of females with gout, there was a trend that females were more likely to suffer from flares (contrast 0.27, 95% CI − 0.01, 0.55, *p* = 0.054). A higher BMI was associated with an increased probability of flares (contrast 0.025, 95% CI 0.003, 0.046, *p* = 0.027), and within participants with flares, a higher probability of ≥ 2 flares (contrast 0.017, 95%CI − 0.001, 0.035, *p* = 0.068). Current allopurinol use, within participants with flares, was most strongly associated with a higher probability of ≥ 2 flares (contrast 0.36, 95% CI 0.13, 0.60, *p* = 0.002).

## Discussion

This study is the first representative population-based study of gout flares. Nearly a quarter of all participants with gout reported two or more flares in the last 12 months, and, contrary to current guidelines, almost half of these participants were not on ULT. Frequent gout flares had a negative effect on physical HRQoL, comparable to that seen with a range of chronic health conditions [30, 31]. The prevalence of ULT (37.1% current, 23% previous use) was consistent with previous studies reporting 28–51% current ULT [7, 9, 22, 32]. Despite the established role of ULT in reducing flares [21], participants on ULT were more likely to experience frequent gout flares, suggesting suboptimal use. Furthermore, the ULT discontinuation rate was nearly 40%. Collectively, these results are consistent with suboptimal management of gout, as has been identified in previous studies [16, 22, 32].

This 2017 representative population-based study demonstrated a high prevalence of self-reported, medically diagnosed gout (6.5%, 95%CI 5.5%, 7.5%) in the South Australian population aged 25 and over. This prevalence is comparable to previous population-based estimates in the South Australian population [4, 33], but greater than the 1.5% prevalence reported from an Australian primary care-based study [34]. It is also higher than prevalence estimates from Europe and America, which range between 0.9 and 3.9% [1, 2, 35]. There is, however, substantial heterogeneity between gout prevalence estimates [36], with case definition identified as an important contributor to this heterogeneity [36]. We used self-reported, medically diagnosed gout for case definition in this study, which has been validated against a hospital discharge diagnosis of gout or use of a gout-specific medication in two American population-based cohorts [37], and shown to have high sensitivity and precision for case definition for gout genetic studies [38]. As case ascertainment through medical records is contingent on both the patient seeking treatment and accurate recording of current and previous diagnoses, case definition by self-reported, medically diagnosed gout will capture a wider spectrum of patients.

There are known difficulties in optimizing ULT for the management of gout. Current rheumatology guidelines recommend a treat-to-target approach, requiring regular serum urate monitoring and slow up-titration of the dose until target serum urate levels are achieved [10–12]. Flares can be precipitated by an initial sudden decrease in serum urate levels and may still occur until all tophi have resolved, which may be some time after the target serum urate level has been reached [39]. While concomitant prophylaxis may prevent this, prescribing is not always appropriate or effective [22]. Subsequently, patients may perceive therapy to be ineffective and continuation rates can be poor [16, 32]. A lack of education for both medical practitioners and patients has been identified as a key barrier for success in establishing and maintaining ULT [13].

We found that younger participants with gout had lower rates of allopurinol continuation and were more likely to have flares, findings that are comparable to those from two retrospective UK general practice database studies of people with incident gout [40, 41]. Importantly, there was no evidence that low SES was a factor in either flares or ULT use; the predominantly public health care system in Australia may mean that this finding is not generalizable to countries with privatized health care systems. However, the roles of BMI and female gender in the management of gout warrant further consideration. In this study, higher BMI was associated with an increased prevalence of frequent flares, yet these patients were no more likely to receive ULT. Higher BMI has been causally linked to increased serum urate levels using a bidirectional Mendelian randomization approach [42], and a predictive model for allopurinol maintenance dose necessary to achieve serum urate target was highly dependent on body weight [43]. Interestingly, a prospective observational study from the US Multiple Risk Factor Intervention Trial database has demonstrated that, in individual patients with gout, there is a positive relationship between changes in BMI and the risk of recurrent gout flares [44], and therefore, weight loss may potentially contribute to gout management.

Although gout predominantly affects men, women were less likely to commence or adhere to ULT and experienced a greater number of flares in our study. Other studies have identified that women with gout have more severe disease with a greater burden of comorbid conditions [45] and poorer ULT adherence [41]. Gender bias in the diagnosis, management, and treatment of chest pain and cardiovascular disease may contribute to poorer outcomes in women (reviewed in [46]). Further research is required as there are limited data about the effect of gender on gout and its influence on management.

There are several limitations of this study. In addition to the use of self-reported, doctor-diagnosed gout, which has been validated for gout case definition [37, 38], flares were also self-reported. Flares may be subject to recall bias, and lower functional health literacy, identified in self-reported medically diagnosed arthritis, including gout, may also affect the responses obtained [47]. A tool for the definition of gout flare for clinical research, which utilizes patient reported flare as one of the criteria, has been validated and published following our data collection [48], so was not used for this study. Dosage and duration of allopurinol use were not quantified, nor were serum urate levels; therefore, adherence and optimization of treatment were only indirectly assessed.

## Conclusion

This is the first community-based study of gout flare, which utilized rigorous sampling methodology to ensure that the data was representative of the general population. We conclude that gout continues to be a prevalent and poorly managed disease, despite readily available treatment. A quarter of participants with gout reported frequent flares that were associated with reduced physical HRQoL. Current allopurinol use was reported by only 51% of participants with frequent gout flares, suggesting undertreated disease and suboptimal use of ULT.

## Additional file

Additional file 1: **Table S1.** Sociodemographic variables as predictors of gout. Analysis was performed by survey-weighted logistic regression, and coefficients represent log-odds ratios. **Table S2.** Prevalence of gout by gender, age, and BMI. **Table S3.** Prevalence of gout % (95%CI) by age × gender. **Table S4.** Sociodemographic variables as predictors of allopurinol use (within gout participants). Analysis was performed by survey-weighted multinomial logistic regression, and coefficients represent log-odds ratios. **Table S5.** Sociodemographic variables and allopurinol use as predictors of the number of gout flares in the preceding year (within gout participants). Analysis was performed by survey-weighted multinomial logistic regression, and coefficients represent log-odds ratios. (PDF 139 kb)

## Data Availability

Data and material available for this study would require further approval upon request from the corresponding author.

## Abbreviations

ACR: American College of Rheumatology
BMI: Body mass index
EULAR: European League Against Rheumatism
HOS: Health Omnibus Survey
HRQoL: Health-Related Quality of Life
IRSAD: Index of Relative Socioeconomic Advantage and Disadvantage
MCS: Mental Component Score
PCS: Physical Component Score
SES: Socioeconomic status
ULT: Urate-lowering therapy
WHO: World Health Organization
