# Supplementary material for: Gout, flares, and allopurinol use: a population-based study
Source: Arthritis Res Ther. 2019 May 31;21:132. doi: 10.1186/s13075-019-1918-7 (PMC6544947; doi:10.1186/s13075-019-1918-7)
Supplement: Supplementary file 1 — Table S1. Sociodemographic variables as predictors of gout. Analysis was performed by survey-weighted logistic regression, and coefficients represent log-odds ratios. Table S2. Prevalence of gout by gender, age, and BMI. Table S3. Prevalence of gout % (95%CI) by age × gender. Table S4. Sociodemographic variables as predictors of allopurinol use (within gout participants). Analysis was performed by survey-weighted multinomial logistic regression, and coefficients represent log-odds ratios. Table S5. Sociodemographic variables and allopurinol use as predictors of the number of gout flares in the preceding year (within gout participants). Analysis was performed by survey-weighted multinomial logistic regression, and coefficients represent log-odds ratios. (PDF 139 kb) [file 13075_2019_1918_MOESM1_ESM.pdf]

Table S1. Sociodemographic variables as predictors of gout. Analysis was performed by survey-weighted logistic regression, and coefficients represent log-odds ratios.

Gout Predictor	Coef.	Std. Err.	t	P>t	-95% CI	+95% CI
Intercept	-2.30	0.15	-15.69	< 0.001	-2.59	-2.01
Sex (base = Male)						
Female	-1.67	0.22	-7.74	< 0.001	-2.09	-1.24
Age[1]						
Linear	5.E-02	1.E-02	5.15	< 0.001	3.E-02	7.E-02
Quadratic	1.E-04	4.E-04	0.29	0.771	-6.E-04	9.E-04
BMI[2]						
Linear	0.12	0.03	4.62	< 0.001	0.07	0.18
Quadratic	-3.E-03	2.E-03	-1.96	0.051	-6.E-03	1.E-05
IRSAD[3]						
Linear	-2.E-03	1.E-03	-2.22	0.027	-5.E-03	-3.E-04
Quadratic	-1.E-05	6.E-06	-1.96	0.050	-2.E-05	6.E-09

[1]Age variable centred around 52
[2]BMI variable centred around 28
[3]IRSAD (SES) variable centred around 970

Table S2. Prevalence of Gout by gender, age and BMI

Group	Gout Prevalence % (95% CI)
Gender:	
Male	10.5% (8.8, 12.5)
Female	2.6% (1.9, 3.6)
Age Group:	
25-34	2.6% (1.3, 5.4)
35-44	2.5% (1.2, 5.4)
45-54	2.8% (1.6. 4.8)
55-64	8.1% (5.6, 11.5)
65+	13.4% (11.3, 15.8)
BMI (WHO)	
Normal/Underweight	2.9% (2.0, 4.2)
Overweight	6.8% (5.3, 8.6)
Obese	11.0% (8.7, 13.9)

Table S3. Prevalence of Gout % (95% CI) by Age x Gender

Age	Gender	
	Male	Female
25-34	4.9% (2.3, 10.4)	0.3% (0.0, 2.4)
35-44	4.4% (1.8, 10.1)	0.7% (0.2, 2.6)
45-54	4.7% (2.6, 8.3)	0.9% (0.2, 4.2)
55-64	13.8% (9.4, 19.9)	2.7% (1.0, 7.3)
65+	21.6% (17.7, 26.0)	6.6% (4.8, 9.0)

Table S4. Sociodemographic variables as predictors of allopurinol use (within gout participants). Analysis was performed by survey-weighted multinomial logistic regression, and coefficients represent log-odds ratios

Allopurinol Predictor	Coefficient	Std.Err	t	P>t	-95% CI	+95% CI
NEVER USE	(base outcome)					
PRIOR USE						
Intercept	-0.90	0.43	-2.12	0.035	-1.74	-0.07
Sex (base = Male)						
Females	-0.71	0.55	-1.29	0.20	-1.79	0.37
Age[3]						
Linear	-3.E-02	2.E-02	-1.35	0.18	-6.E-02	1.E-02
Quadratic	2.E-03	8.E-04	2.44	0.015	4.E-04	4.E-03
BMI[2]						
Linear	4.E-02	5.E-02	0.85	0.40	-5.E-02	1.E-01
Quadratic	-2.E-03	3.E-03	-0.75	0.45	-7.E-03	3.E-03
IRSAD[3]						
Linear	-3.E-03	3.E-03	-1.00	0.32	-8.E-03	3.E-03
Quadratic	9.E-06	2.E-05	0.58	0.56	-2.E-05	4.E-05
CURRENT USE						
_cons	0.24	0.39	0.62	0.54	-0.53	1.01
Sex (base = Male)						
Females	-2.28	0.51	-4.48	< 0.001	-3.28	-1.28
Age[1]						
Linear	7.E-03	2.E-02	0.32	0.75	-4.E-02	5.E-02
Quadratic	6.E-04	9.E-04	0.69	0.49	-1.E-03	2.E-03
BMI[2]						
Linear	3.E-02	4.E-02	0.68	0.50	-5.E-02	1.E-01
Quadratic	-2.E-03	3.E-03	-0.83	0.41	-7.E-03	3.E-03
IRSAD[3]						
Linear	-7.E-04	3.E-03	-0.28	0.78	-6.E-03	4.E-03
Quadratic	-1.E-05	2.E-05	-0.76	0.45	-5.E-05	2.E-05

[1]Age variable centred around 52

[2]BMI variable centred around 28

[3]IRSAD (SES) variable centred around 970

Table S5. Sociodemographic variables and allopurinol use as predictors of the number of gout flares in the preceding year (within gout participants). Analysis was performed by survey-weighted multinomial logistic regression, and coefficients represent log-odds ratios

Flares Predictor	Coef	Std.Eff	t	P>t	-95% CI	+95% CI
**FLARES = 0**	(base outcome)					
**FLARES = 1**						
Intercept	-0.49	0.50	-0.98	0.33	-1.48	0.49
Allopurinol Use (Base = Never)						
Prior	-0.61	0.68	-0.89	0.37	-1.94	0.73
Current	-1.06	0.58	-1.85	0.065	-2.19	0.07
Sex (base = Male)						
Female	0.51	0.63	0.80	0.42	-0.73	1.74
Age[1]						
Linear	-0.048	0.021	-2.28	0.023	-0.089	-0.007
Quadratic	-8.E-04	1.E-03	-0.79	0.43	-3.E-03	1.E-03
BMI[2]						
Linear	0.033	0.067	0.50	0.62	-0.099	0.165
Quadratic	-8.E-04	3.E-03	-0.25	0.80	-7.E-03	5.E-03
IRSAD[3]						
Linear	3.E-03	4.E-03	0.76	0.45	-4.E-03	1.E-02
Quadratic	2.E-05	2.E-05	1.21	0.23	-2.E-05	6.E-05
**FLARES >=2**						
Intercept	-1.22	0.52	-2.32	0.02	-2.24	-0.19
Allopurinol Use (base = Never)						
Prior	-0.16	0.67	-0.23	0.82	-1.47	1.16
Current	0.89	0.51	1.72	0.085	-0.12	1.90
Sex (base = Male)						
Female	1.09	0.52	2.08	0.038	0.058543	2.11363
Age[1]						
Linear	-0.021	0.020	-1.05	0.30	-0.060	0.018
Quadratic	-5.E-04	8.E-04	-0.63	0.53	-2.E-03	1.E-03
BMI[2]						
Linear	0.18	0.06	2.92	0.004	0.06	0.30
Quadratic	-1.E-02	5.E-03	-2.04	0.042	-2.E-02	-3.E-04
IRSAD[3]						
Linear	1.E-05	3.E-03	0.00	1.00	-6.E-03	6.E-03
Quadratic	5.E-06	1.E-05	0.31	0.76	-2.E-05	3.E-05

[1]Age variable centred around 52

[2]BMI variable centred around 28

[3]IRSAD (SES) variable centred around 970
